# Single-molecule characterization of Fen1 and Fen1/PCNA complexes acting on flap substrates

**DOI:** 10.1093/nar/gkt1116

**Published:** 2013-11-13

**Authors:** Timothy D. Craggs, Richard D. Hutton, Alfonso Brenlla, Malcolm F. White, J. Carlos Penedo

**Affiliations:** ^1^SUPA, School of Physics and Astronomy, University of St Andrews, St Andrews, Fife, KY16 9SS, UK and ^2^Biomedical Sciences Research Complex, University of St Andrews, St Andrews, Fife KY16 9SS, UK

## Abstract

Flap endonuclease 1 (Fen1) is a highly conserved structure-specific nuclease that catalyses a specific incision to remove 5′ flaps in double-stranded DNA substrates. Fen1 plays an essential role in key cellular processes, such as DNA replication and repair, and mutations that compromise Fen1 expression levels or activity have severe health implications in humans. The nuclease activity of Fen1 and other FEN family members can be stimulated by processivity clamps such as proliferating cell nuclear antigen (PCNA); however, the exact mechanism of PCNA activation is currently unknown. Here, we have used a combination of ensemble and single-molecule Förster resonance energy transfer together with protein-induced fluorescence enhancement to uncouple and investigate the substrate recognition and catalytic steps of Fen1 and Fen1/PCNA complexes. We propose a model in which upon Fen1 binding, a highly dynamic substrate is bent and locked into an open flap conformation where specific Fen1/DNA interactions can be established. PCNA enhances Fen1 recognition of the DNA substrate by further promoting the open flap conformation in a step that may involve facilitated threading of the 5′ ssDNA flap. Merging our data with existing crystallographic and molecular dynamics simulations we provide a solution-based model for the Fen1/PCNA/DNA ternary complex.

## INTRODUCTION

The activity of Flap Endonuclease 1 (Fen1) as a divalent metal ion-dependent phosphodiesterase is essential to maintain genome integrity in all domains of life (1,2). As a central component of the DNA replication and repair mechanisms, Fen1 recognizes and removes bifurcated RNA or DNA junctions known as 5′ flaps in a sequence-independent manner (3,4). In humans, 5′ flaps are generated 5 million times per cell cycle during lagging strand DNA replication and failure to eliminate them would compromise cell viability (5,6). In DNA repair processes, Fen1 is required for non-homologous end joining of double-stranded DNA breaks and for long-patch base-excision repair (lpBER) (1,2,7). Consistent with this critical role of Fen1 preventing genome instability, mutations that decrease expression levels or alter biochemical activity predispose humans and mouse models to a number of genetic diseases and cancer (5,6). Biochemical and structural studies of Fen1 proteins from phage to humans have shown that members of the FEN family have activity on a variety of branched DNA structures (1–4); however, the optimal substrate leading to efficient catalysis differs among species (1–4,7). For instance, a 5′ double flap containing a 3′ unpaired nucleotide is the optimal substrate for Fen1 endonucleases from archaeal and eukaryotic organisms (8), whereas phage Fen1s are known to prefer pseudo-Y structures (7). The mechanism by which the presence of this 3′-extrahelical nucleotide enhances the catalytic rate and promotes Fen1 cleavage exactly 1 nt into the downstream duplex has received considerable attention (1–4,6,9–11). Recent crystal structures of archaeal Fen1 in complex with dsDNA carrying a 3′-overhang (11) and human Fen1 in complex with a double-flap substrate (12) provided a general model to rationalize the FEN family’s specificity (1–3). Structure-specific recognition of double-flap substrates arises from a combination of sharp bending of the flexible junction using two separate DNA binding sites and specific interactions of the 3′ unpaired nucleotide with a cleft adjacent to the upstream dsDNA binding site (12,13). In fact, Fen1 enclosing a single 3′ nucleotide ensures the cleavage product is ready for ligation and also directs the 5′-ssDNA flap through a conserved helical arch using a threading mechanism, thus solving a highly debated question regarding Fen1 engagement with 5′ flaps (3,13).

In addition to the enhancement of Fen1 activity by the presence of the 3′ unpaired nucleotide (12,13), it is also known that Fen1 association with the proliferating cell nuclear antigen (PCNA) stimulates Fen1 function *in vitro* by up to 50-fold, depending on the experimental conditions (14). The archaeal/eukaryotic PCNA, the prokaryotic β-clamp and the Rad9-Hus1-Rad1 (9-1-1 complex) are some examples of these multimeric toroidal structures that encircle duplex DNA and coordinate DNA processing (15). The role of sliding clamps as coordinators of cellular machineries that act in DNA replication, DNA repair and DNA modification together with their ability to enhance the activity of a variety of DNA-processing enzymes has long been recognized (16–18). However, whether sliding clamps act only as landing platforms where proteins can dynamically exchange during DNA processing (18), or whether they play a more active role remains poorly understood. Potentially, enhancement of protein function by sliding clamps can take place at several steps of the DNA-processing cycle. Protein activation may involve facilitating recruitment to the DNA-editing site, enhancing recognition of the DNA substrate or by directly participating in the catalytically competent complex, as recently found for the *Sulfolobus solfataricus* Xeroderma Pigmentosum Group F endonuclease (XPF) (19,20). Remarkably, despite the ever-increasing number of proteins that have been shown to directly interact with PCNA and for which such interaction is known to have functional consequences (15–19), there is currently very little information regarding PCNA-activation mechanisms. Despite PCNA’s moderate effect on Fen1 activity *in vitro*, disruption of this interaction in a mouse model resulted in slow cell proliferation and embryonic lethality (21,22). In fact, PCNA accompanies Fen1 in most Fen1-involved cellular pathways suggesting a crucial role for the PCNA/Fen1 complex (1,14–18,22). In general, the interaction between PCNA and its client proteins, including Fen1, is highly conserved and takes place between the PCNA-interacting motif (PIP-box) in the client protein and the interdomain connector loop (IDC) of a PCNA subunit (14–18). The trimeric architecture of the PCNA ring can accommodate distinct replication and repair partners simultaneously competing for PCNA subunit association (19,22,23). Indeed, the crystal structure of human Fen1 with PCNA revealed three nuclease proteins bound to the sliding clamp (22) and recent *in vitro* reconstitution of the Okazaki fragment maturation complex from crenarchaeon *S. solfataricus* supports a model in which a single-PCNA ring acts as the assembly platform for Fen1, the DNA polymerase PolB1 and the ATP-dependent DNA ligase Lig1 (18). Although the crystal structures of *S. solfataricus* PCNA on its own (23) and in a complex with Fen1 (24) are available, no solution-based model of the Fen1/PCNA/DNA ternary complex has been reported.

In this study, we have used a combination of Förster resonance energy transfer (FRET) and protein-induced fluorescence enhancement (PIFE) at ensemble and single-molecule level to investigate the activation mechanism of *S. solfataricus* Fen1 by the sliding clamp PCNA and propose a nuclease-reaction profile. Our data suggest a model in which in the absence of Fen1, the double-flap DNA exhibits a Mg^2+^-dependent fluctuation between a Y-shape structure (in the absence of Mg^2+^) and a more extended duplex conformation. Fen1 binding significantly distorts the overall structure of the DNA flap, inducing a pronounced opening of the flap structure at the branch point, as observed in the x-ray crystal structure. PCNA association increases Fen1 affinity for the flap structure without altering the catalytic step and additionally cooperates with Fen1 to promote the flap opening/threading step. Finally, we use our solution-based FRET and PIFE data to refine current models of the Fen1/PCNA/DNA ternary complex based on existing crystal structures and molecular dynamics (MD) simulations.

## MATERIALS AND METHODS

### Oligonucleotide labelling and purification

Oligonucleotides were purchased from Integrated DNA Technologies either unlabelled, or labelled with Cy3/Fluorescein and/or an internal amino modifier C6-dT. Succinimidyl ester derivatives of the fluorophores Cy3, Cy5 (GE Healthcare) and Alexa 488 (Invitrogen) were used according to the manufacturer’s protocol for the specific labelling of DNA oligonucleotides. The various double-flap substrates were assembled using 0.1 OD of each of the relevant strands (Supplementary Materials and Methods section and Supplementary Table S1) and mixed with hybridization buffer (20 mM Tris–HCl pH 7.8, 25 mM NaCl). Samples were then heated at 93°C for 2 min followed by slow overnight cooling to 4°C.

### Protein expression and purification

*Sulfolobus solfataricus* Fen1 was expressed as described previously (20) and purified using a HiLoad® 26/60 Superdex® 200 gel filtration column (GE Healthcare, see Supplementary Material for details). *Sulfolobus solfataricus* PCNA heterotrimer subunits were expressed and purified as described previously (25).

### PCNA labelling

The point mutation N131C (see Supplementary Figure S1) was introduced to the PCNA1 expression plasmid by QuickChange (Agilent Technologies), and the mutant protein was expressed and purified as per the wild type. Pure protein was conjugated to Cy5-maleimide (GE Healthcare) according to the manufacturer instructions. Briefly, the protein was incubated in 50 mM Tris–HCl buffer, pH 7.2, 500 mM NaCl, 10-fold molar excess of Cy5 maleimide (GE healthcare) for 1 h at room temperature. Reaction was stopped with 10 mM dithiothreitol (DTT). Cy5 labelled enzyme was separated from the free dye using two successive PD-10 columns. Labelling yield (∼80%) was assessed by UV-VIS absorbance spectra, correcting for the dye absorbance at 280 nm.

### Molecular modelling of the DNA/Fen1/PCNA complex

Molecular models were built in Pymol starting from a MD simulation derived model of the human DNA/Fen1/PCNA complex (26) and mean dye positions were modelled by the accessible volume (AV) approach using software from the Seidel lab (27). See ‘Supplementary Materials and Methods section’ for detailed description.

### Ensemble fluorescence experiments

Ensemble experiments were performed in 30 mM HEPES, pH 7.6, 40 mM KCl, 5% glycerol, 0.1 mg/ml bovine serum albumin with 5–50 nM DNA substrate. For experiments performed in the presence of PCNA, addition of the clamp loader RFC was not required as PCNA can readily diffuse on to the short synthetic DNA substrates used in this study. Experiments were performed using a Cary Eclipse spectrofluorimeter (Varian Inc., Palo Alto, CA, USA), equipped with a Peltier temperature controller set to 20°C as described in ‘Supplementary Materials and Methods’ section.

### sm-FRET measurements

Single-molecule FRET (sm-FRET) trajectories were acquired from immobilized single-molecules using a prism-type total-internal reflection setup based on an inverted microscope as described in the Supplementary Methods section.

### Measurement of cleavage activity

A flow cell was constructed by affixing plastic tubing to a drilled polyethylenglycol (PEG) passivated quartz slide (28). The unattached end of one piece of tubing was placed in an eppendorf containing buffer. The open end of the other tubing was attached to a syringe, allowing buffer to be drawn through the flow cell by suction. Control experiments to confirm that the loss of Cy5 emission was not due to photobleaching were performed using the Cy5 direct excitation method (See Supplementary ‘Materials and Methods’ section).

## RESULTS

### Global structure and dynamics of 5′-flap DNA substrates

**Figure 1.**
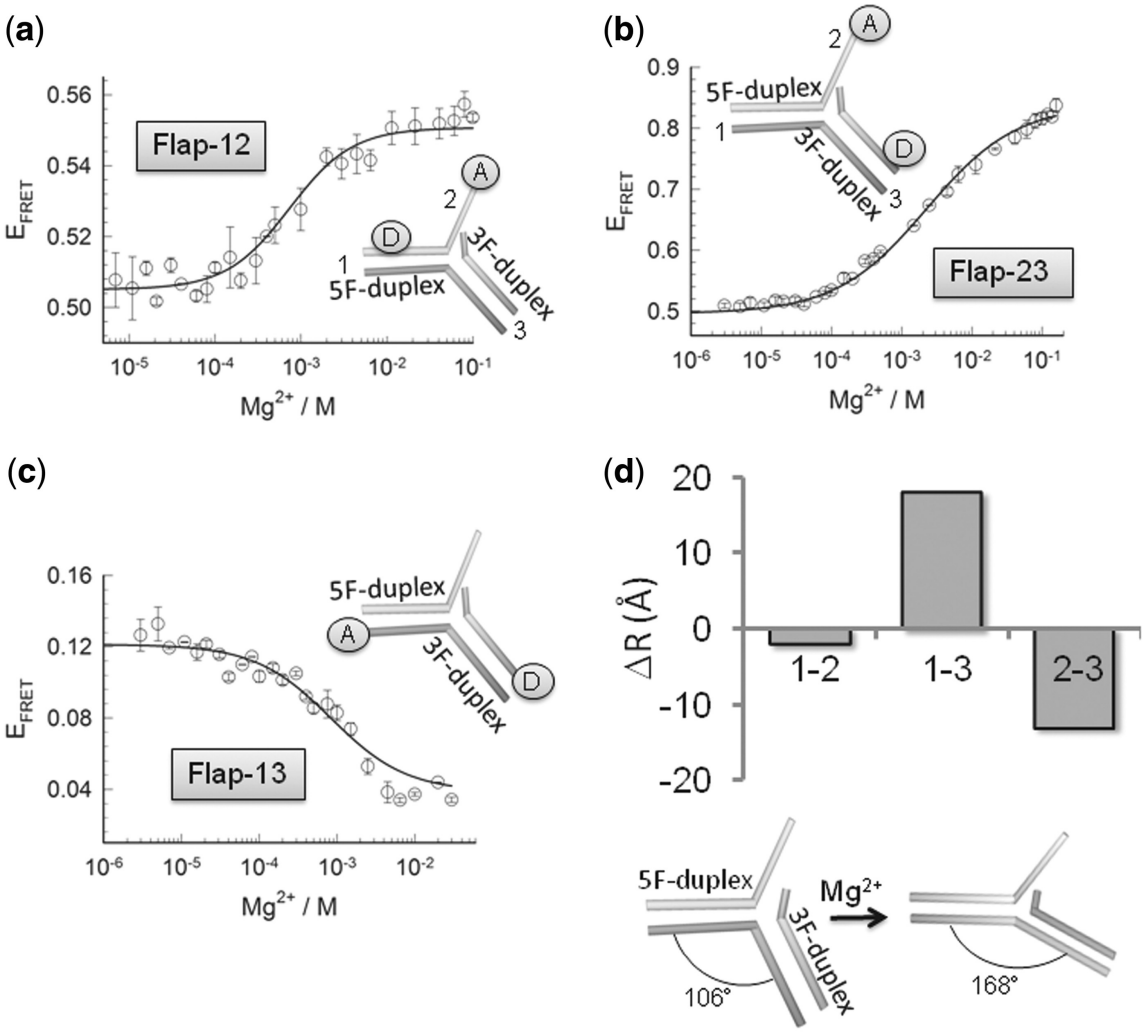
Conformational changes on the double-flap DNA structure induced by the addition of Mg^2+^ ions. Intra-molecular FRET assay to monitor the effect of Mg^2+^ ions with flap substrates labelled with donor and acceptor at the indicated positions (insets). Variation in FRET efficiency as a function of Mg^2+^ ions at 20°C on Flap-12 (**a**), Flap-23 (**b**) and Flap-13 (**c**). Individual fitting of each FRET isotherm for each flap substrate using a two-state model described by Supplementary Equation S1 (see Supplementary ‘Materials and Methods’ section) are shown as continuous lines. Experiments were performed in triplicate and error bars represent the SEM. (**d**) (Upper panel) Comparison of the relative changes in inter-dye distance (Å) observed for the three 5′-flap substrates upon addition of Mg^2+^ ions. (Lower panel) Diagram showing the overall angle changes in flap structure induced by Mg^2+^ ions.

Despite the fact that crucial cellular process including DNA replication and lpBER generate 5′-flap DNA substrates as intermediate products (1,11,12,29), the global structure and dynamics of these bifurcated moieties in the absence of processing proteins is largely unknown. Here, we used intra-molecular FRET to investigate the structure of unbound 5′-flap DNA substrates as a function of Mg^2+^ ions. Three FRET constructs were engineered carrying the donor and the acceptor dye at different positions (Figure 1 and Supplementary ‘Materials and Methods’ section). The double-flap substrate contains two duplex regions that we have termed the 5′-flap-duplex (5F-duplex which refers to the duplex containing the 5′-flap strand) and the 3′-flap-duplex (3F-duplex, which refers to the duplex region containing the 3′-flap strand, see Figure 1). Flap-12 and Flap-23 report structural distortions involving the 9-nt single-strand flap and the 5F-duplex (Flap-12) or the 3F-duplex (Flap-23), while Flap-13 reports changes in the kink angle between the two duplex regions. On addition of magnesium ions, all three flap constructs exhibited significant variations in the efficiency of energy transfer (E_FRET_), indicative of a global reorganization of the flap structure (Figure 1a–c). All conformational changes took place in the mM range of Mg^2+^ ion concentration and were well fitted by a two-state model (Supplementary Equation S1). Indeed, a global fit of the three flap vectors yielded a good fit of all the experimental data with a value of *n* = 0.96 for the Hill coefficient and a half-point of [Mg^2+^]_1/2_ = 1.14 mM (*R* = 0.998) (Supplementary Table S2).

We next analysed in detail the relative variation in FRET efficiencies and inter-dye distances induced by the addition of Mg^2+^ ions (Figure 1d, Supplementary Table S3 and Supplementary Methods section for details regarding the calculation of FRET-derived distances). For Flap-12 and Flap-23, the FRET efficiency increased from 0.51 ± 0.04 and 0.50 ± 0.01, in the absence of Mg^2+^, to values of 0.56 ± 0.02 and 0.84 ± 0.01 at 20 mM concentration of Mg^2+^ ions, respectively. This implies a moderate 2 Å decrease in distance for Flap-12 and ∼13 Å for Flap-23 (Figure 1d, upper panel, Supplementary Table S3). In contrast, Flap-13 exhibited a decrease in FRET efficiency from a value of 0.13 ± 0.01 with no Mg^2+^ ions to a value of 0.034 ± 0.004 in the presence of 30 mM Mg^2+^ ions concentration, which represents a ∼18 Å decrease in the inter-dye distance (Figure 1d upper panel, Supplementary Table S2). A significant shortening of the end-to-end distance associated with to Flap-13 can be explained by a change in the kink angle between the upstream and downstream complexes centred at the phosphate opposite the ss/dsDNA junction. Using the calculated inter-dye distance for Flap-13 and taking into account the length of the fluorescein linker and that Cy3 stacks at 5 Å distance from the terminal base pair of duplex DNA (30), we estimated a decrease in the kink angle from 106° in the absence of Mg^2+^ ions to 168° at 30 mM Mg^2+^ (Figure 1d, bottom panel).

**Figure 2.**
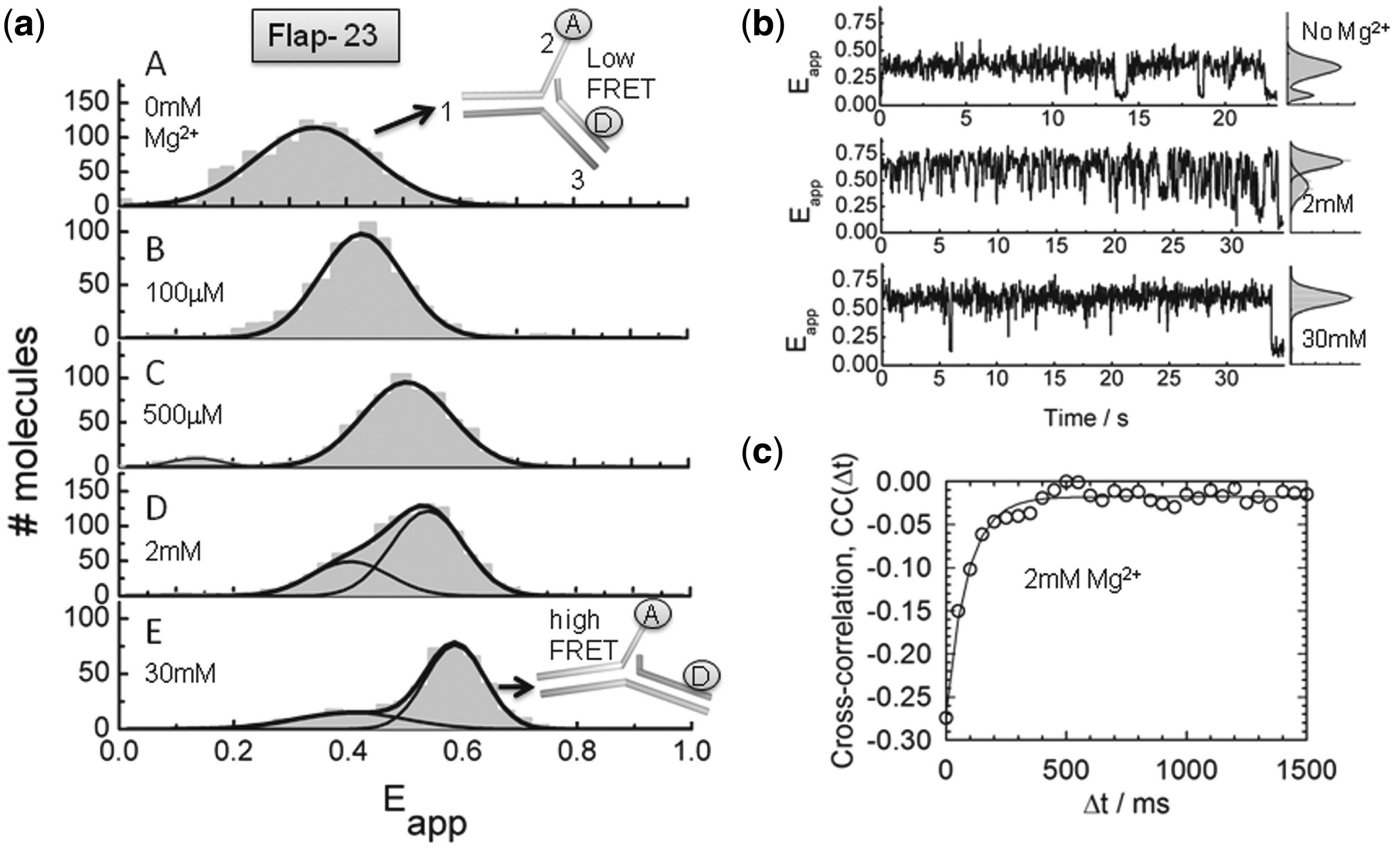
Structural transitions in the Flap-23 substrate studied by sm-FRET. (**a**) Histograms of FRET efficiency summed over multiple single molecules in the absence (panel A) and presence of increasing Mg^2+^ ion concentrations (panels B–D). Individual traces were filtered to remove contributions from fluorophore blinking and photobleaching. (**b**) Representative sm-FRET trajectories (33 ms integration time) as function of Mg^2+^ ion concentration. Corresponding FRET histograms for each trajectory are shown as panels on the right. (**c**) Donor and acceptor intensities were obtained with 16 ms integration time at 2 mM Mg^2+^ ion concentration and the cross-correlation signal was calculated and averaged for 15 traces. Solid line represents the fitting to a single-exponential decay to extract the sum of the backward and forward rates.

In view of the potential for structural heterogeneity in solution and to get insights into the dynamics of flap substrates, we carried out sm-FRET experiments using immobilized Flap-23 constructs. Flap-23 is well-suited for sm-FRET studies because of its high variation in FRET efficiency as a function of Mg^2+^ ions (Figure 1b). Single-molecule time traces and apparent FRET efficiency (*E*_app_) histograms were obtained for different Mg^2+^ ion concentrations (Figure 2a and b and Supplementary Figure S2). In the absence of Mg^2+^ ions, the flap substrate remained in a low-FRET state (*E*_app_ ∼ 0.37) until photobleaching occurred (Figure 2a, panel A and Figure 2b, upper trace). As the concentration of Mg^2+^ ions is increased, the single-molecule histograms showed a predominant Gaussian peak that progressively shifted to higher FRET (Figure 2a, panels B–D), reaching a value of *E*_app_ ∼ 0.6 at 30 mM concentration of Mg^2+^ (Figure 2a, panel E). Interestingly, at concentrations of Mg^2+^ ions between 1 and 30 mM, the single-molecule histograms also showed a minor contribution of an additional Gaussian peak with a similar *E*_app_ (∼0.37) to that obtained in the absence of Mg^2+^. At these conditions, the single-molecule trajectories exhibited fast, but clear transitions between these two FRET states. Because the observed fluctuations were too fast to be clearly resolved using Hidden Markov modelling methods, we performed cross-correlation analysis to determine their rates (Figure 2c) (31). The correlation time (τ) at 2 mM Mg^2+^ ions was extracted from the single-exponential fitting of the cross-correlation curve providing a value of 11.3 ± 2 s^−^^1^ for the rate of fluctuations on the Flap-23 substrate. Taken together, our data suggest a Mg^2+^-induced conformational change taking place on the flap substrate from a bent to an extended structure. Interestingly, at concentrations of Mg^2+^ ions where Fen1 exhibits maximal catalytic activity (∼10 mM) (32), we observed the flap substrate alternating rapidly between both structures, whereas at concentrations of Mg^2+^ ions known to inhibit Fen1 function (>30 mM), the flap substrate remained locked in the extended conformation. The fast inter-conversion observed here for the flap substrate using sm-FRET is in good agreement with recent studies on the structure of nicked, gapped and bulged structures, which have shown that these intermediates are highly dynamic and able to adopt a broad range of conformations (33–35).

### Fen1 directs PCNA loading to the 3F-duplex region of the double flap

**Figure 3.**
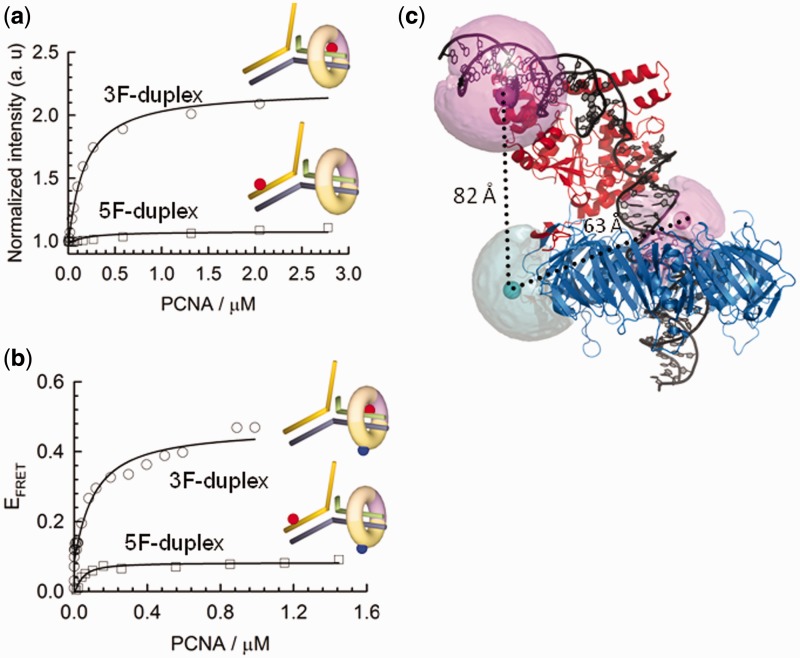
Organization of the ternary complex studied by FRET and PIFE. (**a**) Normalized PIFE of Cy3 emission in a background of 10 mM Ca^2+^ and 5 µM Fen1 obtained for Cy3-labelled upstream and downstream duplexes as a function of PCNA concentration. Data were fitted to a binding model as described by Supplementary Equation S3. (**b**) Variation in inter-molecular FRET efficiency between an upstream or downstream Cy3 donor-labelled DNA flap and Cy5 acceptor-labelled PCNA as a function of PCNA concentration. (**c**) Modelling of the ternary Fen1:DNA:PCNA complex using MD simulation derived from (26) and the FRET distances extracted in (b). Pink and cyan spheres represent mean dye positions modelled using the AV approach (27,37) (See ‘Materials and Methods’ section and Supplementary Material for details).

To shed light on the organization of the Fen1/PCNA complex bound to a double-flap substrate, we determined the position of the PCNA ring relative to the double flap in the presence of Fen1. We previously demonstrated that PCNA on its own encircles the 3F-duplex (*K*_D_ = 8.5 µM) and 5F-duplex (*K*_D_ = 6.95 µM) regions with equal probability and with a 1:1 stoichiometry (20). Here, we used a different approach based on PIFE, in which a fluorescent dye becomes brighter when a protein binds in close proximity (36). This photophysical property, exhibited by Cy3 and other dyes has been previously employed to explore the positioning of sequence-specific *BamH*I restriction enzymes along duplex DNA (36). We designed double-flap DNA substrates with a 9-nt 5′-ssDNA flap carrying an internal Cy3 fluorophore positioned either 10-nt downstream or upstream of the 5′-flap junction (Figure 3a) and performed PCNA titrations in a 5-µM background of Fen1 and in the presence of 10 mM concentration of Ca^2+^ ions to prevent cleavage. As demonstrated for other metal ion-dependent nucleases, Ca^2+^ ions efficiently stabilized the nucleic acid–protein interaction but did not support catalysis (20,23). For the 5F-duplex Cy3-labelled flap substrate pre-incubated with Fen1, only a small increase in Cy3 fluorescence emission (∼10%) was detected. In contrast, the 3F-duplex labelled substrate showed a 2-fold relative increase in fluorescence emission and a *K*_D_ of 160 ± 14 nM (Figure 3a). This *K*_D_-value for PCNA interacting with the Fen1/DNA complex represents a 50-fold decrease in dissociation constant when compared to DNA alone and compares favourably with previous Fen1/PCNA dissociation constants (∼210 nM) (38). These data confirm that in the Fen1/PCNA/DNA ternary complex, PCNA loads onto the 3F-duplex dsDNA region that is below the 5′ ssDNA flap as previously predicted using a streptavidin-biotin blocking assay (39) and recently proposed from MD simulations (26). For comparison, and given that PCNA is an heterotrimer in which PCNA1 and PCNA2 subunits form an stable heterodimer (PCNA12) to which PCNA3 binds; we carried out similar experiments with the tightly associated PCNA12 complex. Interestingly, a substantial 45% increase in fluorescence emission was still observed using the 3F-duplex Cy3-labelled substrate and we obtained a *K*_D_ of 360 ± 30 nM for the PCNA12/Fen1 interaction. A moderate decrease (∼2-fold) in the affinity of PCNA12 when compared to the heterotrimeric PCNA provides strong experimental evidence for selective recruitment of Fen1 to PCNA12 but not PCNA3 as observed from the crystal structure of the PCNA12/Fen1 complex (40). Also, the additional contacts lost in the absence of PCNA3 may account for the observed decrease in affinity.

### Organization of the Fen1/PCNA/DNA ternary complex

FRET studies to investigate the structural organization of the eukaryotic Fen1/PCNA/DNA complex in solution are challenging due to the homotrimeric nature of the sliding clamp, which facilitates binding of up to three copies of the same client protein, as observed in the crystal structure of Fen1 with PCNA (24). Here, we took advantage of the heterotrimeric nature of the *S. solfataricus* PCNA (23,25) to circumvent these problems and obtain sliding clamps singly labelled at the PCNA1 subunit. For this, a PCNA1 variant carrying a single-cysteine mutation (N131C) was labelled with a thiol-reactive maleimide derivative of Cy5 and incubated with unlabelled subunits PCNA2 and PCNA3 to reconstitute the sliding clamp (See Supplementary ‘Materials and Methods’ section). Titration of acceptor-labelled PCNA (PCNA-Cy5) with upstream or downstream donor-labelled DNA substrates, identical to those used for the PIFE experiments, and pre-equilibrated with 5 µM Fen1, revealed a significantly higher intermolecular FRET efficiency (Supplementary Equation S2) for the 3F-duplex labelled substrate (*E*_app_ ∼ 0.48) corresponding to a distance of 63 ± 5 Å, compared to the 5F-duplex labelled complex (*E*_app_ ∼ 0.12, 82 ± 7 Å) (Figure 3b). A shorter distance from PCNA to the 3F-duplex dsDNA region agrees with our previous observations using PIFE (Figure 3a) and confirms PCNA loading to the 3F-duplex region of the flap substrate in the presence of Fen1. The dissociation constant recovered from these experiments (*K*_D_ of 117 ± 34 nM) also matches that obtained by fluorescence enhancement using non-labelled PCNA, indicating that the presence of Cy5 acceptor on the PCNA does not affect the formation of the ternary complex.

Recently, a structural model of the human Fen1/PCNA/DNA ternary complex was reported (26). This was constructed by combining all available high-resolution crystal structures of the individual components and subassemblies, and then refined by multi-nanosecond atomistic MD simulations. Using our experimental FRET data, we were able to assess whether this model was consistent with the ternary complex structure present in solution. To compare FRET-derived inter-dye distances with those derived from atomic structures, it is important to correctly model the dye positions with respect to their attachment points on the biomolecules. To do this, we used an AV approach which takes into account the various dimensions of the dye and linker (37,41; see ‘Materials and methods’ section for details), to model the mean dye positions onto the ternary structure (Figure 3c). The resulting distances from PCNA to the 3F-duplex (60 Å) and 5F-duplex regions (83 Å) are in excellent agreement with our FRET-derived distances, thus providing the first experimental evidence in support of this ternary complex structure. It is also interesting to note that the AV of the Cy3 dye located at the 3F-duplex is significantly restricted by the PCNA ring (Figure 3c), which is consistent with the increased PIFE effect seen for this labelling position (Figure 3a).

### Affinity of Fen1 and Fen1/PCNA complexes for flap DNA substrates

**Figure 4.**
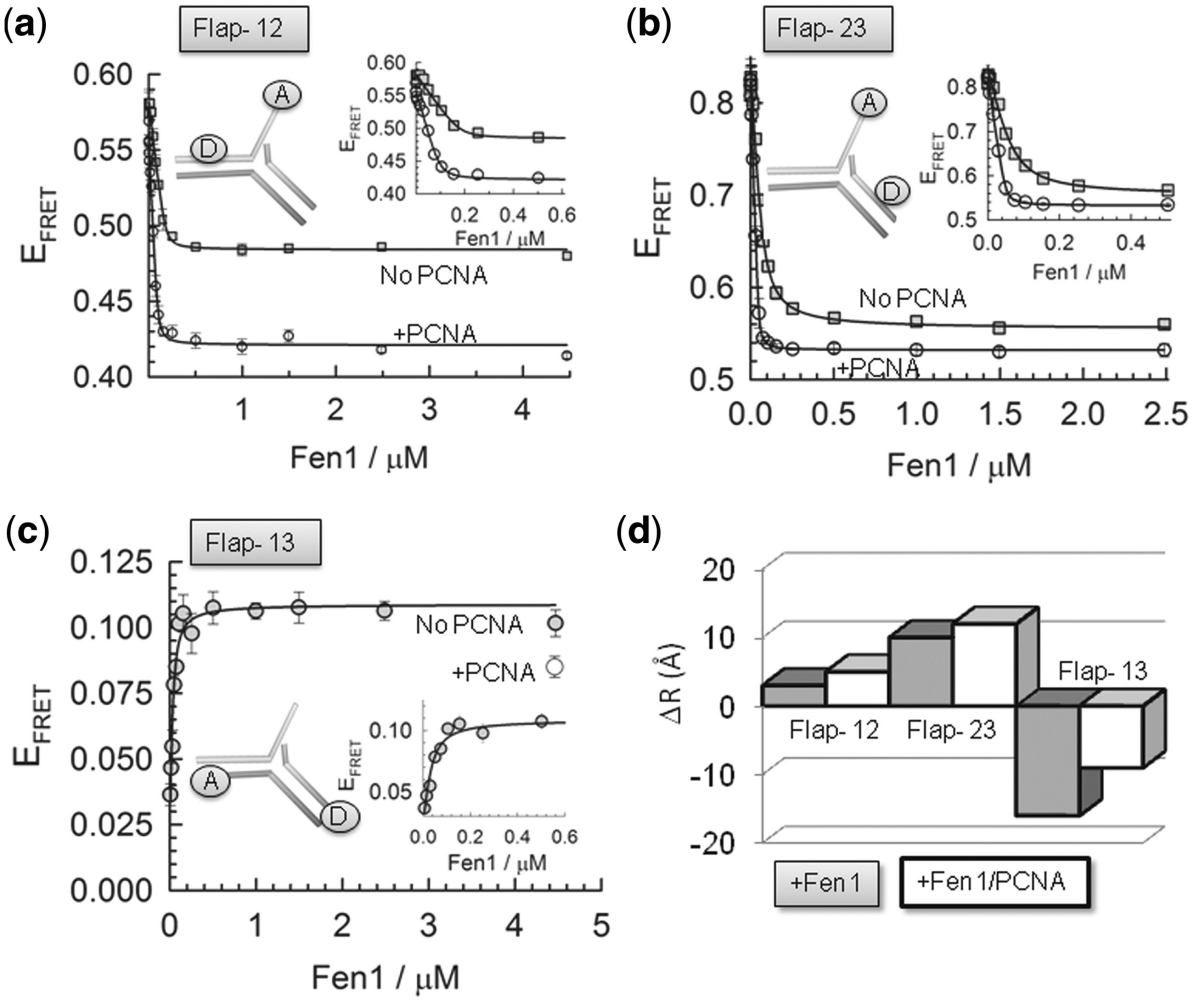
DNA distortion by Fen1 in the absence (squares) and presence (circles) of PCNA at 10 mM Ca^2+^ ion concentration and 20°C. Intra-molecular FRET was used to monitor the interaction between Fen1 and different flap substrates (**a**) Flap-12, (**b**) Flap-23 and (**c**) Flap-13. Solid lines represent the fitting to the FRET-binding isotherm for each flap using a binding model described by Supplementary Equation S3. (**d**) Comparison of the relative changes in inter-dye distances (Å) observed for the three 5′-flap substrates upon association to Fen1 (grey) and Fen1/PCNA complexes (white).

Once the structures and dynamics of the flap DNA substrate in the unbound state and the organization of the ternary complex were determined, we investigated Fen1 association to the 9-nt ssDNA 5′ flap in the absence and presence of PCNA (2 µM). FRET-binding curves were obtained at room temperature by titrating each vector with increasing concentrations of Fen1 in a background of 10 mM Ca^2+^ ions to prevent cleavage (Figure 4a–c, Supplementary Table S4). Global fitting of the three binding curves to Supplementary Equation S3 yielded a *K*_D_ of 14 ± 3 nM that fitted accurately (*R* = 0.998) all Fen1 binding isotherms (Figure 4a–c). This value is very similar to that reported for Fen1 binding to flap substrates using electrophoretic mobility assays (*K*_D_ ∼ 5–10 nM), suggesting that the presence of the fluorophores has no significant effect on complex formation (38,39). FRET titrations performed under identical conditions, but in the presence of 2 µM PCNA (Figure 4a–c), revealed a decrease in the dissociation constant (*K*_D_ ∼ 3 ± 1 nM). This result is in good agreement with the 4-fold reduction in *K*_M_ observed at room temperature under steady-state conditions using a similar flap substrate (38). For comparison, a 5′-flap DNA substrate lacking the 3′ extra-helical nucleotide yielded a *K*_D_ in excess of 10 µM in the absence of PCNA, that was only moderately rescued (*K*_D_ ∼ 0.7 ± 0.1 µM) in the presence of 2 µM PCNA (Supplementary Figure S3).

### Reorganization of the flap substrate by Fen1 and Fen1/PCNA binding

Previous studies using FRET provided evidence that *Archaeoglobus fulgidus* Fen1 promotes a bent conformation between the 5F-duplex and 3F-duplex regions (11); however, the extent to which the DNA-flap structure bound to Fen1 is affected by the presence of PCNA is still currently unknown. Therefore, we used a similar FRET approach to determine the global reorganization taking place in the flap DNA structure induced by Fen1 and Fen1/PCNA complexes. In the absence of PCNA, addition of Fen1 altered the overall structure of the flap substrate (Figure 4d and Supplementary Table S5). At saturating concentrations of Fen1 (>1 µM), the inter-dye distance associated with Flap-12 increased only by ∼3 Å, whereas the equivalent duplex DNA to single-strand distance reported by Flap-23 exhibited a 10 Å increase in the presence of Fen1 (Figure 4d). For Flap-13, the end-to-end distance between the 5F-duplex and 3F-duplex regions decreased by 16 Å upon Fen1 binding, which is consistent with earlier reports and with the X-ray crystal structure. We next repeated these experiments in a background of 2 µM PCNA. Because addition of PCNA induces as a fluorescence enhancement of the Cy3 FRET donor located in the 3F-duplex (Figure 3a), the inter-dye distances were corrected following procedures described in the literature (20) (see Supplementary Equation S4). For Flap-12 and Flap-23, addition of PCNA promoted an additional increase in the inter-dye distance of ∼2 Å (Figure 4d). For Flap-13 the inter-dye distance increased by ∼8 Å in the presence of PCNA (Figure 4d).

### Single-molecule assay to monitor Fen1/PCNA substrate recognition

Based on recent crystallographic data (3,11,12,42), opening of the Flap-23 vector has emerged as a characteristic feature shared by FEN superfamily endonucleases as a way to target specific DNA structures. Experimental evidences for a Fen1-induced double-nucleotide unpairing mechanism being responsible for the observed opening of the Flap-23 vector have been recently reported in two elegant studies using 2-aminopurine fluorescence (43) and disulfide cross-linking between base pairs either side of the scissile phosphate (44). The pronounced variation in FRET efficiency observed for Flap-23 (ΔE_FRET_ ∼ 0.3) upon Fen1 and Fen1/PCNA binding allowed us to use sm-FRET to test this flap opening model in the presence and absence of the sliding clamp. A double-flap substrate (Flap-23) carrying a 9-nt 5′-ssDNA flap, similar to that used for ensemble-FRET measurements, was modified for sm-FRET using the donor–acceptor FRET pair Cy3-Cy5 (*R*_o_ ∼ 53 Å) (28). A biotin moiety was also incorporated at the 5′ end of the downstream duplex for surface immobilization to a PEG functionalized quartz microscope slide via a neutravidin–biotin interaction (28).

**Figure 5.**
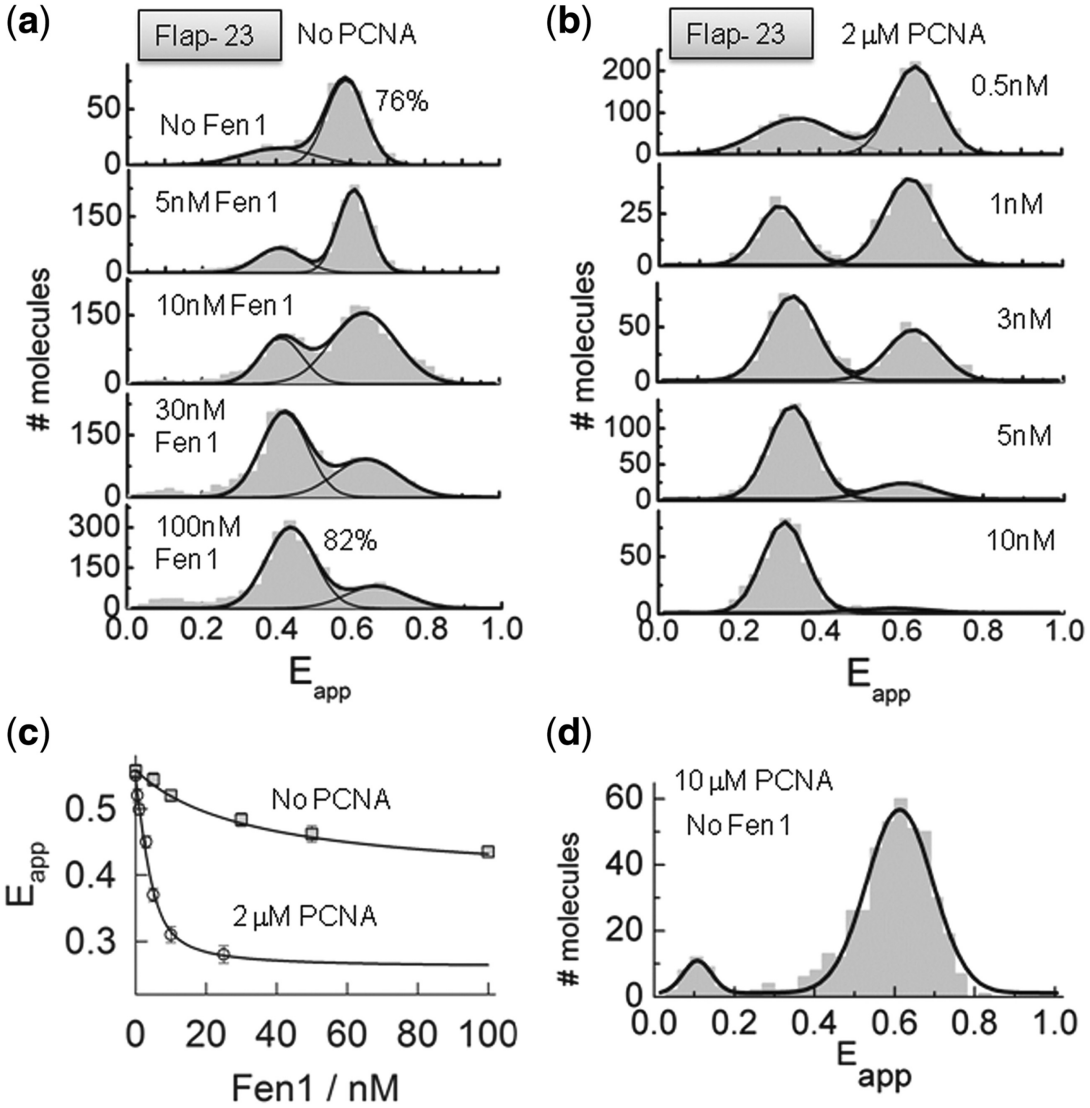
Opening of the flap substrate by Fen1 and Fen1/PCNA complexes studied using sm-FRET. sm-FRET histograms obtained for surface-immobilized Flap-23 substrates in the absence (**a**) and presence of 2 µM PCNA (**b**). Gaussian fits corresponding to the unbound and bound species are also shown. (**c**) Variation in averaged FRET efficiency integrated from the sm-FRET histograms shown in (a) and (b) as a function of Fen1 concentration in the absence (squares) and presence of 2 µM PCNA (circles). Solid lines represent the fit to a binding model as described in Supplementary Equation S3. (**d**) sm-FRET histogram and corresponding Gaussian fit obtained for Flap-23 in the presence of 10 µM PCNA.

The variation in FRET efficiency of surface-immobilized flap substrates (Flap-23) was monitored as a function of Fen1 concentration (Figure 5a). In the absence of Fen1 protein, the sm-FRET histogram displayed two unbound states with Gaussian peaks centred at *E*_app_ ∼ 0.62 ± 0.1 (76%) and a minor contribution at *E*_app_ ∼ 0.38 ± 0.2 (Figure 5a, top panel). As previously shown (Figure 1), these two FRET populations can be assigned to the extended and bent conformations of the flap substrate, the latter being the predominant conformer at 10 mM concentration of Ca^2+^ ions. As the concentration of Fen1 protein was increased, the sm-FRET histograms showed a progressive decrease in the contribution of the high-FRET population (*E*_app_ ∼ 0.65 ± 0.2) and a concomitant increase in the contribution of the low-FRET state (*E*_app_ ∼ 0.44 ± 0.2), reaching a value of 82% at 100 nM concentration of Fen1 (Figure 5a, bottom panel). Judging by the relative similarity of their FRET values, Fen1 binding to the DNA substrate seems to stabilize Flap-23 into a conformation (*E*_app_ ∼ 0.44 ± 0.2) very close to that observed in the absence of Mg^2+^ ions (*E*_app_ ∼ 0.37 ± 0.1) (Figure 1). However, both conformational states displayed very different dynamic properties (see later), which allowed us to assign them unambiguously. Thus, we interpreted the single-molecule population centred at low-FRET (*E*_app_ ∼ 0.44 ± 0.2) as arising from Fen1–DNA complexes in which Fen1 binding to the flap substrate increases the inter-dye distance as previously found using ensemble FRET (Figure 4b and d).

The ability of single-molecule methods to monitor with high accuracy the formation of very high affinity protein–DNA complexes prompted us to re-evaluate the values previously obtained for the dissociation constant using ensemble-averaging methods. For this, we extracted the average FRET value from the sm-FRET histograms obtained at each Fen1 concentration and the results were fitted to a similar model as previously described (see Supplementary ‘Materials and Methods’ section) (Figure 5c, No PCNA). We obtained a dissociation constant of 23 ± 2 nM in the absence of PCNA, which is in very good agreement with the value calculated using ensemble FRET (14 ± 3 nM). This agreement also provides additional evidence to confirm that surface immobilization of the flap substrate via its 5F-duplex does not disrupt the Fen1–DNA interaction.

In order to study the effect of PCNA on Fen1 binding to the flap substrate and the subsequent opening of the Flap-23 vector, a sm-FRET titration of surface-immobilized substrates at increasing concentrations of Fen1 protein was repeated in a background of 2 µM PCNA and 10 mM Ca^2+^ (Figure 5b). However, prior to studying the Fen1:PCNA:DNA ternary complex at single-molecule level, we performed control experiments to investigate the possibility of PCNA inducing a conformational change on the flap substrate in the absence of Fen1 protein. sm-FRET histograms in the presence of 10 µM PCNA showed a single-Gaussian peak centred at an identical FRET efficiency value to that observed in the absence of PCNA (*E*_app_ ∼ 0.6 ± 0.2) (Figure 5d), therefore confirming that PCNA alone does not disrupt the flap structure. As we increased the concentration of Fen1, the contribution of the high-FRET peak corresponding to the unbound state progressively decreased and shifted to a low-FRET value (*E*_app_ ∼ 0.32 ± 0.08). This low-FRET population became the predominant contribution at concentrations of Fen1 higher than ∼3 nM (∼65%) and therefore can be assigned to the formation of the PCNA:Fen1:DNA ternary complex. From these data it is clear that the presence of PCNA had two major effects in the mechanism of Fen1 recognition of the flap substrate. First, it increased Fen1’s affinity for the flap substrate. Indeed, average FRET values extracted at each Fen1 concentration from the sm-FRET histograms and fitted to the model described by Supplementary Equation S3 provided a *K*_D_-value of 1.6 ± 0.5 nM (Figure 5b) which is more than 10-fold lower than in the absence of PCNA. This increase in affinity agrees with the proposed role of PCNA recruiting Fen1 to the flap junction. Secondly, a shift to lower FRET (*E*_app_ ∼ 0.32 ± 0.08) in the ternary complex, compared to Fen1 alone (*E*_app_ ∼ 0.44 ± 0.2), confirmed the moderate increase in the inter-dye distance observed in ensemble data (Figure 4b and d).

### Equilibrium dynamics of Fen1 and Fen1/PCNA complexes bound to surface-immobilized substrates

**Figure 6.**
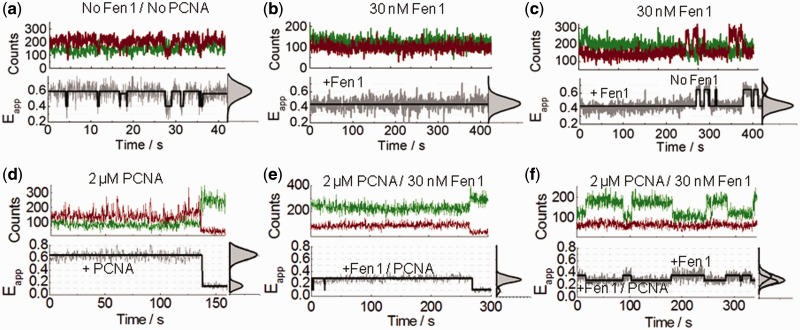
Equilibrium dynamics of Fen1 and Fen1/PCNA-induced flap opening using sm-FRET. (**a–c**) Representative single-molecule intensity traces of donor (green) and acceptor (red) intensities and corresponding FRET traces (grey) obtained for Flap-23 in a background of 10 mM Ca^2+^ ions concentration in the absence (a) and presence (b, c) of 30 nM Fen1. (**d–f**) Representative single-molecule time records of donor (green) and acceptor (red) intensities and corresponding sm-FRET traces (grey) obtained for Flap-23 in a background of 10 mM Ca^2+^ ion concentration and 2 µM PCNA, in the absence (d) and presence (e, f) of 30 nM Fen1. Solid black lines represents the estimated trajectory obtained using Hidden Markov Modelling. FRET histograms and corresponding Gaussian fits for each trace are also shown in the right panels.

To get some insights into the binding dynamics of Fen1 to flap DNA, we monitored the donor and acceptor intensities from surface-immobilized Flap-23 substrates for extended periods in a background of 10 mM Ca^2+^ to avoid cleavage. Representative time-intensity traces and corresponding FRET trajectories in the absence and presence of Fen1 are shown in Figure 6. In the absence of Fen1, the trajectories displayed flap substrates remaining for several tens of seconds in the extended state (*E*_app_ ∼ 0.62 ± 0.2) with occasional short-lived fluctuations (<1s) to the bent conformation (*E*_app_ ∼ 0.37 ± 0.2) (Figure 6a). In contrast, when the flap substrate was pre-incubated with progressively higher concentrations of Fen1, an increasing percentage of traces remained in a low-FRET state (*E*_app_ ∼ 0.44 ± 0.2) for long periods of time (∼5–8 min) before photobleaching (Figure 6b and Supplementary Figure S4). Because the probability of these long-lived low-FRET trajectories increased with the concentration of pre-incubated Fen1, we assigned them as representing Fen1/DNA bound complexes exhibiting a very low dissociation dynamics. At each Fen1 concentration analysed, a small subset of these low-FRET trajectories (<15–20%) displayed very occasional transitions between both FRET states that we interpreted as Fen1 binding/unbinding events (Figure 6c and Supplementary Figure S4). The statistical frequency of these fluctuations remained low even when the observation time was significantly increased (>15 min). We additionally confirmed that the slow dissociation kinetics did not result from surface-immobilization artefacts using an ensemble-FRET competition assay (Supplementary Figure S5). To get additional insights into the effect of PCNA, we next investigated the single-molecule equilibrium dynamics of flap substrates incubated with Fen1 in background of 2 µM PCNA and 10 mM Ca^2+^. Although the lack of a significant PCNA-induced effect on the predominant structure of the flap substrate was already confirmed (Figure 5d), and before studying the effect of PCNA/Fen1 complexes, we decided to further test whether PCNA alone could affect the dynamics of the flap substrate (Figure 6d). We observed that addition of 2 µM concentration of PCNA has a subtle effect on the flap dynamics with most of the trajectories lacking the occasional fast transitions to the low-FRET state (*E*_app_ ∼ 0.37) observed for the flap alone (Figure 6a). We propose that in the absence of Fen1 directing PCNA loading to the 3F-duplex region, PCNA can randomly assembly at each side of the flap junction and freely scan each of the duplex regions. Thus, the observed PCNA-induced subtle stabilization of the extended flap conformer, which is predominant at 10 mM Ca^2+^, may arise from a restricted ability of the flap substrate to adopt the bent conformation, most likely due to the steric hindrance provided by the freely diffusing sliding clamp.

In the presence of Fen1/PCNA complexes, no statistically significant transitions to a high-FRET state (*E*_app_ ∼ 0.6), corresponding to Fen1/PCNA dissociation events, were detected during the time scale of our observation window (∼5 min) (Figure 6e–f, Supplementary Figure S6). Surprisingly, a high percentage (>70%) of the single-molecule trajectories showed a non-anticorrelated behaviour where the donor emission fluctuated between two intensity levels, differing by ∼2-fold; while the acceptor intensity remained practically unchanged (Figure 6f, Supplementary Figure S6). As a result, the corresponding FRET traces displayed transitions between efficiency values of *E*_app_ ∼ 0.3 and *E*_app_ ∼ 0.44. Taking into account that the two-state dynamics were only detected for Fen1/DNA complexes in the presence of PCNA and that a 2-fold fluorescence enhancement (PIFE) of the Cy3 donor located in the 3F-duplex was already confirmed by ensemble measurements (Figure 3a), we interpreted this as arising from binding events of PCNA to Fen1/DNA complexes. An alternative explanation involving the transient association of PCNA3 (*K*_D_ ∼ 1 µM) to PCNA12 permanently bound to the Fen1/DNA complex, was ruled out because a control experiment using only PCNA12 exhibited a similar non-anticorrelated dynamics (Supplementary Figure S7). Overall, the invariance of the Cy5 acceptor signal can be explained by the formation of the Fen1/PCNA/DNA complex leading to two competing processes that counteract one another. PCNA binding promotes an increase in quantum yield of the Cy3 donor in the 3F-duplex, which would normally lead to a concomitant increase in acceptor signal; however, this effect is almost entirely compensated for by a simultaneous PCNA-induced opening of the Flap-23 distance. Such an increase in inter-dye distance leads to a less efficient energy transfer process to the acceptor dye and therefore lower Cy5 fluorescence. The fact that this increase in distance in the ternary complex compared to Fen1/DNA has been observed in both, ensemble (Figure 4b and d) and single-molecule experiments (Figure 5d and 6f), using two different FRET pairs, is strong evidence that we are observing a PCNA-induced conformational change.

### Single-molecule nuclease-reaction profiles

**Figure 7.**
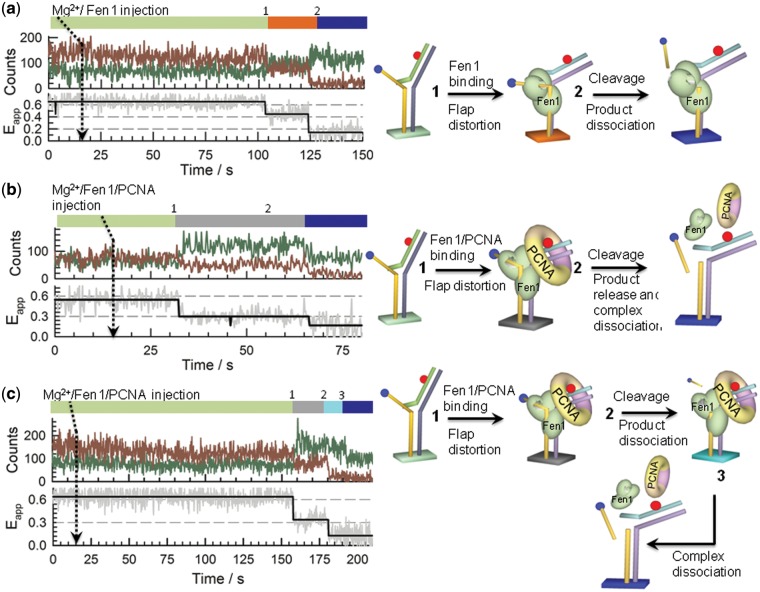
Single-molecule Fen1 nuclease-activity profiles. Dissecting binding and cleavage events was performed on surface-immobilized Flap-23 substrates using real-time injection of Fen1 or pre-incubated Fen1/PCNA complexes while monitoring the donor (green) and acceptor (red) intensity trajectories. (**a**) Representative single-molecule reaction profile obtained after real-time injection of Mg^2+^/Fen1 indicated by an arrow at 15 s. Sequential binding and cleavage events were revealed by a sharp transition (1) from high- (*E*_app_ ∼ 0.6) to low-FRET (*E*_app_ ∼ 0.44) followed by a loss of the Cy5 signal due to cleavage and release of the 5′-flap ssDNA region into the solution (2), respectively. (**b**) Representative single-molecule reaction profile obtained after real-time injection of Mg^2+^/Fen1/PCNA indicated by an arrow at 15 s. As before, Fen1/PCNA binding was revealed by a sudden decrease in FRET (1), but now to a lower FRET value (*E*_app_ ∼ 0.3). Subsequent loss of the Cy5 signal due to cleave of the 5′-flap ssDNA region is shown in (2) together with the Cy3 signal decreasing due to PCNA-induced PIFE. (**c**) A small percentage of Fen1/PCNA reaction profiles (∼10%) revealed binding (1), cleavage and product release (2) and a third event (3) representing PCNA molecules that remained bound to the nicked product for some period of time (∼15 s in the trace shown). After this interval, PCNA dissociated from the nicked substrate leading to a decrease in Cy3 emission due to the lack of PIFE effect.

We used real-time injection experiments to monitor Fen1 nuclease activity at single-molecule level. Because the acceptor dye is located at the 5′ end of the single-strand flap, cleavage of this single-stranded region in the Flap-23 substrate should lead to a complete loss of the FRET signal, while the formation of Fen1/DNA (*E*_app_ ∼ 0.44) or Fen1/DNA/PCNA (*E*_app_ ∼ 0.32) complexes can be easily distinguished by their distinctive FRET levels as shown in the previous sections. Representative real-time single-molecule trajectories and corresponding FRET traces obtained at each condition are shown in Figure 7 and Supplementary Figures S8 and S9.

After injection of Fen1 and 10 mM Mg^2+^ to a flow cell pre-incubated with 10 mM Mg^2+^, most of the Flap-23 substrates remained in the high-FRET unbound state (*E*_app_ ∼ 0.6) for a variable period of time (25–500 s) before the FRET signal abruptly decreased to *E*_app_ ∼ 0.4–0.45. This change in FRET corresponds to the formation of the Fen1/DNA complex (Step 1 in Figure 7a) with the donor and acceptor signals showing a clearly anti-correlated behaviour (Figure 7a, left panel). In the majority of traces (∼80%) this step was followed by a sudden loss of the Cy5 fluorescent signal (Step 2 in Figure 7a) and the simultaneous recovery of the Cy3 emission with practically the same amplitude (Figure 7a). We interpreted this as evidence for Fen1-induced cleavage of the flap substrate and subsequent product dissociation and diffusion into the solution (Figure 7a, right panel). To confirm unambiguously that the observed loss of FRET was indeed caused by Fen1 cleavage of the flap substrate and not simply by photobleaching of the Cy5 acceptor, several control experiments were performed using direct Cy5 excitation methods (see Supplementary ‘Materials and Methods’ section and Supplementary Figure S10).

Similar experiments injecting a pre-incubated Fen1/PCNA complex exhibited a similar pattern to that observed with no PCNA; however, now the FRET signal changed from *E*_app_ ∼ 0.6 (unbound state) to *E*_app_ ∼ 0.3, confirming the assembly of the ternary complex (Figure 7b, left panel). The likelihood that this sudden change in FRET efficiency was caused by Fen1/PCNA binding to the flap (Step 1 in Figure 7b) was also supported by the increase in total fluorescence intensity arising from the expected enhancement in Cy3 quantum yield upon PCNA binding. With our integration time (200 ms), none of the single-molecule trajectories showed transitions involving sequential binding of Fen1 first (*E*_app_ ∼ 0.4), followed by PCNA assembly (*E*_app_ ∼ 0.3). Interestingly, the majority of reaction profiles analysed for the Fen1/PCNA/DNA ternary complex (∼90%) showed a quenching in Cy3 emission simultaneous to the cleavage event (Step 2 in Figure 7b, left panel). This is in marked contrast to the anti-correlated behaviour observed in Fen1/DNA complexes where cleavage of the ssDNA 5′ flap carrying the Cy5 acceptor was simultaneously followed by the recovery of the Cy3 emission (Figure 7a, left panel). The non-anticorrelated cleavage pattern found for these Fen1/PCNA complexes can be explained assuming all steps following the Fen1/PCNA binding event and including cleavage, product release and Fen1/PCNA dissociation, occurred simultaneously (Step 2 in Figure 7b, right panel). In this case, cleavage and break of the energy transfer to the acceptor, which should lead to an enhanced Cy3 emission, was at least partially counteracted by PCNA dissociation and the subsequent lack of the PIFE effect on the Cy3 donor induced by PCNA. These two competing effects produced an overall increase in Cy3 signal, suggesting that in the pre-cleaved ternary complex, the PIFE channel has a dominant contribution to the total Cy3 output. Additionally, a small fraction of the reaction profiles (<10%) showed a non-synchronized cleavage and Cy3-quenching events (Steps 2 and 3 in Figure 7c, left panel). Here, a significant decrease in Cy3 emission was only detected several seconds later than the cleavage step (∼15 s in Figure 7c) suggesting that PCNA remained bound to the nicked substrate after cleavage took place. Interestingly, for these trajectories, no increase in Cy3 emission was observed concomitant with the cleavage and loss of Cy5 emission event (Step 2, Figure 7c). We interpret this as evidence for a subtle rearrangement of the bound complex following product release. Such structural reorganization may change the local environment reducing the PIFE effect around the Cy3 probe and thus counteracting the expected increase in Cy3 emission. It is worth to mention that PIFE is very sensitive to small distances and it has been proposed as a molecular ruler complementary to FRET to detect changes in distance below the 1–2 nm FRET limit (36). Although with our current assay we cannot clarify whether Fen1 remained associated to the cleaved substrate and the statistics of these events was too low to extract any quantitative information, the observation of an stable PCNA/DNA interaction in the nicked product agrees with the proposed role of PCNA acting as a protein-recruitment platform during flap DNA processing (15–17).

**Figure 8.**
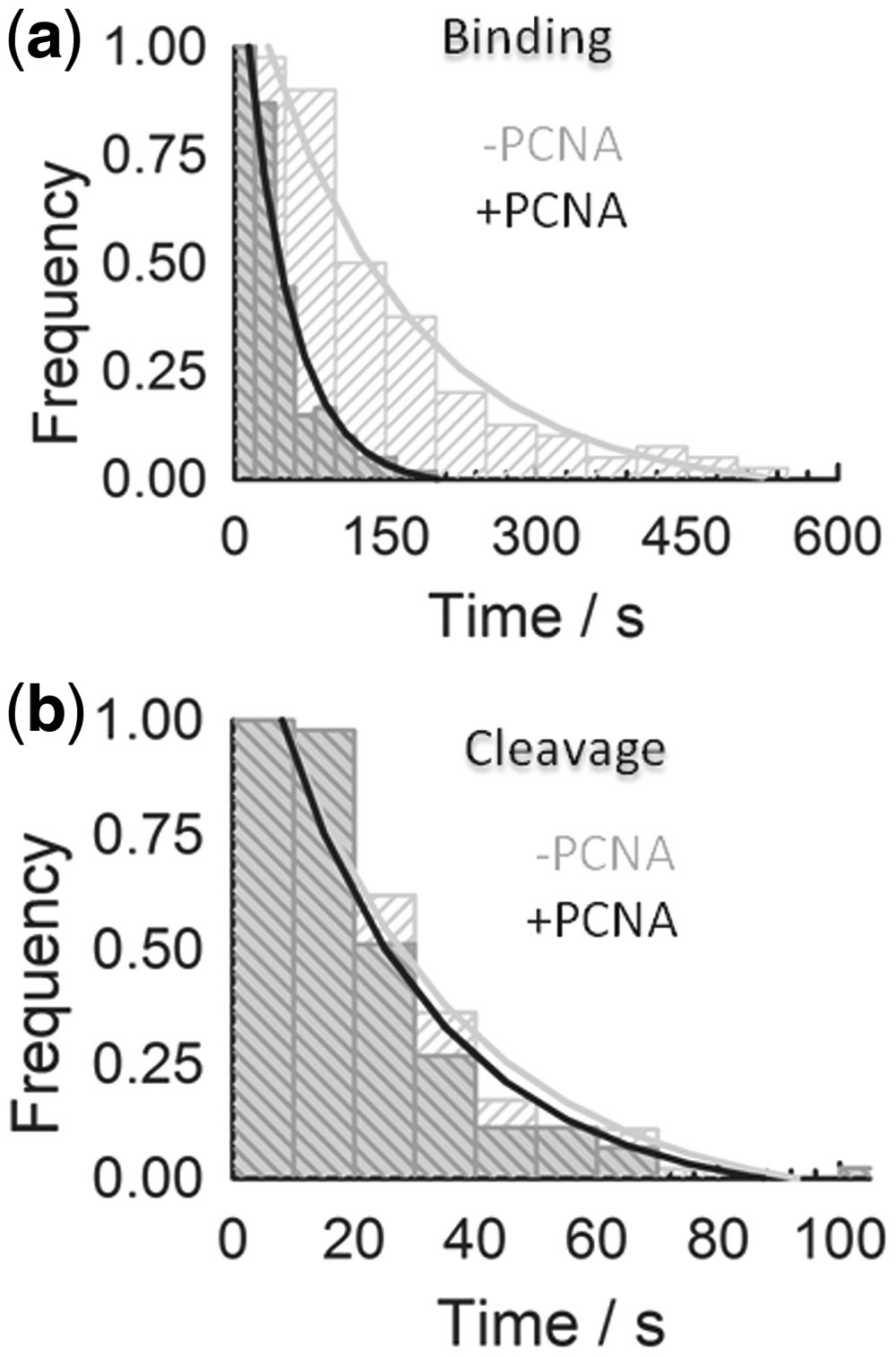
PCNA association enhances Fen1 binding to the substrate but has no effect on the catalytic step. Dwell times for the binding (**a**) and cleavage (**b**) events were directly extracted from the single-molecule reaction profiles in the absence (light grey) and presence of PCNA (dark grey). Dwell times were fitted to single-exponential decay functions to extract the pseudo first-order association rate constants (s^−1^) (a) and the cleavage-product dissociation rate constants (s^−1^) (b).

Using the ability of sm-FRET to differentiate all the Fen1/PCNA processing steps, we have quantified the Fen1 binding (Figure 8a and b) rates separately from the cleavage and product dissociation rates (Figure 8c and d), in the absence and presence of PCNA. For each single-molecule reaction profile, Fen1 and Fen1/PCNA binding rates were extracted by measuring the time interval from the injection of the protein(s) to the appearance of the change in FRET signal from the high-FRET unbound state to the low-FRET bound state. Similarly, the cleavage and product dissociation rates were obtained by measuring the time interval from the binding event to the loss of Cy5 emission. Single-molecule frequency histograms for each of these intervals were plotted and fitted to monoexponential decay functions to extract the corresponding rates. For Fen1 binding to Flap-23, we obtained a pseudo first-order rate constant *k*_obs_ = 0.006 ± 0.002 s^1^ without PCNA and *k*_obs_ = 0.023 ± 0.003 s^1^ in the presence of 1 µM PCNA. For the cleavage rate that included the catalytic and product dissociation steps, we obtained similar values of 0.040 ± 0.002 s^−^^1^ and 0.038 ± 0.004 s^−^^1^ with (1 µM PCNA) and without PCNA, respectively. These values obtained at 20°C are very similar to those previously reported by us (0.022 s^−^^1^) at 25°C using an ensemble fluorimetric assay to monitor cleavage (38) and confirm that PCNA activation of Fen1 takes place exclusively at the level of substrate recognition with no direct effect on the catalytic rate.

## DISCUSSION

**Figure 9.**
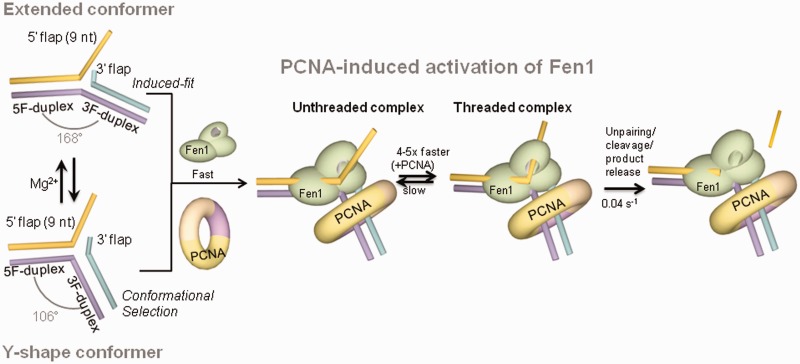
Model of PCNA activation of Fen1-substrate recognition. In the absence of protein, the substrate exhibits an Mg^2+^-dependent equilibrium between a Y-shaped structure and extended conformation with a [Mg^2+^]_1/2_ = 1.2 mM. Binding of Fen1 to the extended conformer induces a kink from 168^°^ to 90–100^°^ between the upstream and downstream duplexes (induced-fit model) and promotes opening of the flap junction. At moderate Mg^2+^ ion concentrations (<2 mM), Fen1 could associate to the already bent Y-shape structure following a conformational selection model. After Fen1 binding to the flap base by establishing interactions with the downstream and upstream duplexes, the flap substrate threads through Fen1’s helical arch. Association and dissociation rates are both slow but in the presence of PCNA, the association rate becomes 4-fold faster, suggesting a role in facilitating the flap threading step.

It has been suggested that Fen1 specificity for certain flap substrates may be linked to their intrinsic flexibility and potential to become distorted by Fen1 (1–3). Despite their prevalence in DNA replication and repair pathways, there is currently very little known about the structure and dynamics of unbound DNA flaps. To address this question, we investigated the structure and flexibility of such flaps, including the effect of divalent metal ions such as Ca^2+^ and Mg^2+^; the latter being indispensable cofactors for Fen1 activity (1) and also well-known folding agents of branched DNA (33–35, 45). Based on our combined ensemble (Figure 1) and sm-FRET data (Figure 2), we introduce a model in which, DNA flaps rapidly fluctuate (*k*_backward_ + *k*_forward_ = 11.3 s^−^^1^) between two conformations; a Y-shape structure and an extended form with a 168° angle between the 5F-duplex and the 3F-duplex (Figure 9); the latter being the predominant structure at high concentrations of divalent metal ions (>30 mM). It is also interesting to note the close positioning of the 9-nt 5′-ssDNA flap to the 3F-duplex in the extended conformation (*E*_app_ = 0.84, Figure 1b) compared to the Y-shape structure (*E*_app_ = 0.5, Figure 1b). By analysing these observations in the context of the known interactions between Fen1 and the DNA flap (1–3,11,12), it is possible to draw some conclusions about the possible role of flap conformation in Fen1’s substrate-recognition step. It has been shown that the substrate binding affinity of murine flap endonuclease reaches an optimal value at ∼5–10 mM Mg^2+^ that rapidly decreases at higher concentrations (32). Although Mg^2+^ ions could affect the structure of Fen1 and therefore its substrate binding efficiency, no reorganization of human Fen1’s local structure was detected using FTIR and SAXS when excess Mg^2+^ was added to the wild-type structure and to the D181A variant, a mutant with similar affinity (*K*_D_ = 7 nM) but unable to cleave (46). Taking these findings together, we propose that Mg^2+^-induced inhibition of Fen1 activity at relatively high concentrations may arise, at least to some extent, from the difficulty to engage with a non-flexible DNA substrate, predominantly locked in an extended conformation, that requires extensive reorganization of its structure to achieve the cleavage-competent form.

Single-molecule experiments revealed the presence of two alternative protein-free structures of the DNA substrate (Figure 2), which also raises the important question of how the recognition process takes place at the molecular level. In agreement with previous studies (11,12), our ensemble-FRET data using the Flap-13 construct (Figure 4c) indicate that Fen1 binds to the DNA substrate with high affinity (*K*_D_ = 14 nM), inducing a pronounced kink (90–100°) at the flap junction between both DNA duplex regions. Based exclusively on these ensemble data, it would be reasonable to conclude that an induced-fit binding model was in operation (47), where protein binding drives the substrate into a more compact conformation (47). However, there is a remarkable similarity between the kink angle of the protein-bound structure (100°) and that calculated by us for Flap-13 (∼106°) in the absence of metal ions, suggesting that the Y-shaped conformer could represent an excellent fit to the Fen1 scaffold. Based on this, and the presence of two rapidly interconverting conformers detected in the single-molecule experiments described here, it seems reasonable to speculate that Fen1 could bind the Y-shaped structure inducing a minimal distortion on the kink angle and displace the equilibrium between both conformations towards the bent structure. A conformational selection model of this type is reminiscent of the interaction mode between the L7Ae protein and equilibrium conformers of the *k*-turn RNA (48). If such conformational selection takes place for Fen1, the protein’s ability to sense the DNA structure would extend beyond detecting the presence of the required DNA binding motifs to also involve testing for their relative conformation. In this context, structure-specific recognition of Fen1 substrates may involve a complex continuum of induced-fit and conformational selection modes (Figure 9). The balance between these recognition mechanisms most likely will be dictated by a delicate interplay between the rate of inter-conversion between conformers of the DNA substrate and the multiple structural and catalytic roles that Mg^2+^ ions seem to hold in Fen1 function (1–3,32,46).

An emerging feature shared across the FEN superfamily of nucleases is their ability to expose the scissile phosphate by opening the junction at the base of the flap and threading the ssDNA portion through a helical gateway that becomes ordered upon threading (1–3,11,12,42). Our ensemble (Figure 4b) and single-molecule data (Figures 5a and 6a–c) on the Flap-23 substrate provide some insights into this mechanism and how this is influenced by PCNA (Figures 5b and 6d–f). In both ensemble- and sm-FRET, we observed a decrease in FRET efficiency for Flap-23 upon addition of Fen1 or Fen1/PCNA. We estimated that this change in FRET efficiency represents an ∼10 Å increase in dye-to-dye distance for Fen1 alone and by ∼12 Å in the presence of Fen1/PCNA. Given that PCNA alone had no effect on the sm-FRET distribution for Flap-23 (Figure 6c), we are confident that the observed distance changes reflect Fen1-induced opening of the DNA junction that is slightly enhanced by PCNA. Our results broadly agree with a recent single-molecule study where a similar increase in FRET induced by Fen1 was observed using flaps of different length (49). However, in contrast to the high dissociation constant reported for Fen1 in that study (1.3 ± 0.3 µM), we obtained a value of 23 ± 2 nM for surface-immobilized Flap-23 substrates (Figure 5b), which is only 3-fold higher than Fen1’s dissociation constant (∼7 nM) previously reported using ensemble methods (46).

To assign the conformational change observed for surface-immobilized Flap-23 substrates upon Fen1 binding to specific molecular-level interactions, we need to take into account the mechanism for Fen1’s recognition of the substrate. According to the threading model, Fen1 establishes contacts primarily to the 5F-duplex via the H2TH motif and then searches for DNA structures that can bend sharply by interacting with the 3F-duplex and the unpaired 3′ nucleotide (3,10–13,46,49). In principle, this range of Fen1 interactions leading to substrate bending and Flap-23 opening could account by themselves for the observed variation in dye-to-dye distance (∼10 Å) without the need to invoke any additional process. However, several lines of evidence suggest that the Fen1-bound Flap-23 substrates showing an *E*_app_ ∼ 0.4 constitute threaded Fen1/DNA complexes (Figure 9) (3,10–13,46). First, the formation of the pre-cleavage complex was detected in real-time as a single-step FRET transition (Figure 7a), suggesting that all significant readjustments of the ssDNA flap occur at the time of binding, at least within our time resolution (200 ms). Secondly, dissociation of Fen1 from a complex where ssDNA flap is threaded through Fen1 may be a slow process due to the difficulty of releasing the trapped flap. This hypothesis was confirmed by the slow dissociation rate measured for Fen1/DNA complexes by ensemble-FRET ((1.33 ± 0.06) × 10^−^^3^ s^−^^1^) and sm-FRET experiments (Figure 6b and c, Supplementary Figure S5), which agrees with previous studies reporting dissociation half-times >10 min for 5-nt ssDNA flaps (39). Interestingly, the pseudo first-order association rate measured using real-time injection (Figure 8a) was also slow (*k*_obs_ = 0.006 ± 0.002 s^−^^1^). It is important to note that in these experiments Fen1 association is reported by flap opening using an intra-molecular FRET assay; thus the measured rate actually represents a combination of the initial binding event, most likely involving interaction of the H2TH domain with the 5F-duplex, plus any subsequent conformational rearrangements taking place on the flap (Figure 9). These slower conformational changes may include Fen1 recognition of the 3′-flap site and bending of the substrate at the flap junction, threading of the ssDNA through Fen1’s cavity, or a combination of both processes. Several studies have reported significantly longer association and dissociation rates for Fen1 as the flap length increased from 2 to 12 nt (39,47). Based on these findings, it has been suggested that the initial Fen1 association step to the flap base is a favourable process regardless of the 5′ ssDNA flap length. In fact, a substrate without 5′ flap showed only a 2-fold increase in *K*_M_, while a single-flap lacking the 3′ unpaired nucleotide increased the *K*_M_ by 8-fold. In contrast, mechanically threading the 5′ flap could present considerable entropic problems that would explain a progressively slower formation of the threaded state as flap length increases (39).

Compared to Fen1 alone, the association rate in the presence of PCNA increased by ∼4–5-fold (Figure 8b), while the catalytic rate remained unchanged (Figure 8d). Assuming a model as described above, in which threading represents the slowest step in the formation of the pre-cleavage complex, we hypothesize that the observed increase in apparent association rate of Fen1 may be indicative of PCNA facilitating the threading process. Whether the exact activation mechanism involves repositioning of Fen1 in the proper orientation to thread the flap, or whether PCNA acts mechanically as a platform from which Fen1 can push the flap more efficiently, will require further clarification. Nevertheless, our findings confirm an active role for PCNA in the formation of the active Fen1-substrate complex that may be shared by other members of the FEN superfamily. In addition to increasing the association rate, real-time cleavage by Fen1 in the presence of PCNA is consistently observed from a ternary complex in which Flap-23 exhibits a lower FRET value (*E*_app_ ∼ 0.3) (Figure 7b and c). This moderate increase in dye-to-dye distance for Flap-23, ∼12 Å compared to ∼10 Å without PCNA, was also detected in ensemble measurements (Figure 3c) and a range of mechanisms may be responsible. Among these, a PCNA-induced kink of the 3F-duplex bound to Fen1 has been suggested by MD simulations of the ternary complex (26) and further confirmed from our FRET data (Figure 3c). Alternatively, an enhanced threading of the ssDNA in the ternary complex may also be possible.

In summary, we demonstrate that unbound flap DNA substrates fluctuate rapidly between a Y-shaped conformer, with a structure relatively close to that observed in complex with Fen1, and an extended conformation (Figure 9). Fen1 binding to these structures could follow either an induced-fit model (predominant at Mg^2+ ^> 2 mM) or presumably a conformational selection model at lower Mg^2+^ ion concentrations. Our work confirms that Fen1 binding to the DNA opens the substrate at the flap base, as seen in the crystal structure, in addition to promoting a kink between the 5F- and 3F-duplex regions of the extended conformer (Figure 9). Fen1’s association and dissociation rates obtained from single-immobilized substrates are slow due to the threading process. We provide the first experimental evidence for PCNA activating by 4–5-fold Fen1’s apparent association rate and subsequent flap threading, without altering catalysis. Given that the interaction of Fen1 and PCNA constitutes a paradigm for PCNA-interacting proteins, our findings establish a framework to further explore PCNA’s role as an architectural organizer of the DNA-processing machinery.

## SUPPLEMENTARY DATA

Supplementary Data are available at NAR Online, including [50–57].
